# Exosomes from human umbilical cord mesenchymal stem cells enhance fracture healing through HIF‐1α‐mediated promotion of angiogenesis in a rat model of stabilized fracture

**DOI:** 10.1111/cpr.12570

**Published:** 2019-01-20

**Authors:** Yuntong Zhang, Zichen Hao, Panfeng Wang, Yan Xia, Jianghong Wu, Demeng Xia, Shuo Fang, Shuogui Xu

**Affiliations:** ^1^ Department of Emergency and Trauma Shanghai Changhai Hospital Affiliated to the Second Military Medical University Shanghai China; ^2^ Department of Orthopaedics and Rehabilitation, School of Medicine Yale University New Haven Connecticut; ^3^ Department of Plastic and Reconstruction Shanghai Changhai Hospital Affiliated to the Second Military Medical University Shanghai China

**Keywords:** angiogenesis, exosomes, fracture healing, HIF‐1α, umbilical cord mesenchymal stem cell

## Abstract

**Objectives:**

Exosomes, as important players in intercellular communication due to their ability to transfer certain molecules to target cells, are believed to take similar effects in promoting bone regeneration with their derived stem cells. Studies have suggested that umbilical cord mesenchymal stem cells (uMSCs) could promote angiogenesis. This study investigated whether exosomes derived from uMSCs (uMSC‐Exos) could enhance fracture healing as primary factors by promoting angiogenesis.

**Materials and Methods:**

uMSCs were obtained to isolate uMSC‐Exos by ultrafiltration, with exosomes from human embryonic kidney 293 cells (HEK293) and phosphate‐buffered saline (PBS) being used as control groups. NanoSight, laser light scattering spectrometer, transmission electron microscopy and Western blotting were used to identify exosomes. Next, uMSC‐Exos combined with hydrogel were transplanted into the fracture site in a rat model of femoral fracture. Bone healing processes were monitored and evaluated by radiographic methods on days 7, 14, 21 and 31 after surgery; angiogenesis of the fracture sites was assessed by radiographic and histological strategies on post‐operative day 14. In vitro, the expression levels of osteogenesis‐ or angiogenesis‐related genes after being cultured with uMSC‐Exos were identified by qRT‐PCR. The internalization ability of exosomes was determined using the PKH67 assay. Cell cycle analysis, EdU incorporation and immunofluorescence staining, scratch wound assay and tube formation analysis were also used to determine the altered abilities of human umbilical vein endothelial cells (HUVECs) administered with uMSC‐Exos in proliferation, migration and angiogenesis. Finally, to further explore the underlying molecular mechanisms, specific RNA inhibitors or siRNAs were used, and the subsequent effects were observed.

**Results:**

uMSC‐Exos had a diameter of approximately 100 nm, were spherical, meanwhile expressing CD9, CD63 and CD81. Transplantation of uMSC‐Exos markedly enhanced angiogenesis and bone healing processes in a rat model of femoral fracture. In vitro, other than enhancing osteogenic differentiation, uMSC‐Exos increased the expression of vascular endothelial growth factor (VEGF) and hypoxia inducible factor‐1α (HIF‐1α). uMSC‐Exos were taken up by HUVECs and enhanced their proliferation, migration and tube formation. Finally, by using specific RNA inhibitors or siRNAs, it has been confirmed that HIF‐1α played an important role in the uMSC‐Exos‐induced VEGF expression, pro‐angiogenesis and enhanced fracture repair, which may be one of the underlying mechanisms.

**Conclusions:**

These results revealed a novel role of exosomes in uMSC‐mediated therapy and suggested that implanted uMSC‐Exos may represent a crucial clinical strategy to accelerate fracture healing via the promotion of angiogenesis. HIF‐1α played an important role in this process.

## Graphical abstract

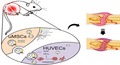

## 1 INTRODUCTION

Fracture healing is a complicated process orchestrated by a precise sequence of growth factors and cytokines that regulate the activation, proliferation and differentiation of local mesenchymal stem or progenitor cells.^1^, ^2^ Approximately 5%‐10% of fractures undergo delayed union or even non‐union, both of which require prolonged or repeated treatments and have a significant impact on treatment cost and quality of life.^1^, ^3^

Transplanted bone marrow mesenchymal stem cells (BMSCs) have been shown to be effective via enhancing osteogenesis and angiogenesis.^4^, ^5^ Nevertheless, these cells’ clinical application is still hampered by the limited sources, painful and invasive harvesting procedures, and safety hurdles.^7^, ^8^ With increasing in‐depth understanding of mesenchymal stem cells in tissue regeneration, an increasing number of reports have implied that the positive effects may be ascribed to the exosomes (Exos) released from mesenchymal stem cells.^9^, ^10^ These 40‐100 nm extracellular vesicles have been demonstrated to have similar effects in various tissues’ regeneration, such as neurites,^13^ myocardia,^14^, ^15^ skin^16^, ^17^ and skeletal muscles^18^ with their derived stem cells. Exosomes are believed to have the ability to regulate cellular activities through transferring certain proteins, genetic information or other molecules to target cells.^19^, ^20^ In addition, the particular lipid bilayer of the exosomal membrane can protect bioactive substances from degradation under adverse conditions, and stable physical and chemical properties make them easy to preserve in vitro.^24^ Therefore, exosomes provide researchers with a novel and promising means of promoting fracture healing.

Umbilical cord mesenchymal stem cells (uMSCs), as an appealing cell source with the advantages of easy collection,^25^ good proliferation and differentiation capacity,^26^ and low immunogenicity,^27^ have been revealed to be effective in bone regeneration via enhancing angiogenesis.^28^ However, studies of uMSC‐derived exosomes (uMSC‐Exos) on bone regeneration have rarely been reported. Considering the therapeutic effects of exosomes similar to those of their parent stem cells, we used uMSCs as cell sources to generate exosomes and investigated whether these exosomes could enhance fracture healing by promoting angiogenesis or in other ways.

In the present study, uMSC‐Exos were transplanted into the fracture site in a rat model of femoral fracture. Enhanced angiogenesis and accelerated bone healing processes were observed. In vitro, other than enhancing osteogenic differentiation, uMSC‐Exos increased the expression of vascular endothelial growth factor (VEGF) and hypoxia inducible factor‐1α (HIF‐1α) and promoted proliferation, migration and tube formation of human umbilical vein endothelial cells (HUVECs). By using specific RNA inhibitors or siRNAs, we have demonstrated that the uMSC‐Exos can change the angiogenic ability of endothelial cells via regulating HIF‐1α, which may be one of the underlying mechanisms to promote fracture healing.

## 2 MATERIALS AND METHODS

### 2.1 Cell culture

Human umbilical cord samples were obtained after healthy neonatal deliveries with permission from the infants’ parents and the Institutional Review Board at Changhai Hospital of the Second Military Medical University. Primary cultures of uMSCs were established based on previous methods.^29^ Only uMSCs in passages 2‐5 were used for experiments.

HEK293 cells were purchased from the American Type Culture Collection (ATCC, Rockville, MD, USA) and maintained in DMEM containing 10% foetal bovine serum (FBS; Gibco, Grand Island, NE, USA) at 37°C in an atmosphere with 5% CO_2_.

### 2.2 Isolation and identification of exosomes

The cell suspension medium was collected every other day, and then, we moved it to cone‐shaped tubes for centrifugation at 300 ***g*** for 10 minutes at 4°C to pellet the cells, and the supernatant was centrifuged at 16 500 ***g*** for 20 minutes at 4°C to further eliminate cellular debris. Next, the supernatant was percolated by a 0.22‐μm filter (Merck‐Millipore, Darmstadt, Germany), and the flow‐through was transferred to new tubes for ultracentrifugation at 120 000 ***g*** for 70 minutes at 4°C in a SW32Ti rotor (Beckman Coulter, Brea, CA, USA) to pellet the exosomes. The supernatant was instantly aspirated upon accomplishing of the first ultracentrifugation and immediately ultracentrifuged once more as previously described. NanoSight, a laser light scattering spectrometer, transmission electron microscopy (TEM) and Western blotting were used to identify exosomes.

### 2.3 In vivo studies

All animal studies were conducted in line with the principles and procedures approved by the Second Military Medical University Committee of Animal Resources, which are based on the International Guiding Principles for Biomedical Research Involving Animals.

#### 2.3.1 Surgical procedures

Forty‐eight 12‐week‐old male Wistar rats (weighing 400‐450 g) received a transverse osteotomy in the middle diaphysis of the right femur, and a 1.5‐mm intramedullary needle was inserted to stabilize the fracture. Details for creating the fracture models have been previously described.^30^ We used HyStem‐HP hydrogel (Catalog: GS315, Glycosan Biosystems, Salt Lake City, UT, USA) as a carrier for delivery of exosomes. This hydrogel is a combination of thiol‐modified hyaluronan, HA, and thiol‐modified heparin, which can be injected and crosslinked in situ.^31^, ^32^ uMSC‐Exos (100 μg/mL) were mixed in hydrogel according to the manufacturer's instructions and injected near the fracture site before closing the incision.^33^ An equal volume of HEK293‐Exos (100 μg/mL) as well as phosphate‐buffered saline (PBS) was mixed in hydrogel and served as the control.

#### 2.3.2 Radiographic analysis of fracture callus formation

Radiographs of bones were acquired through a soft X‐ray device (CMB‐2; SOFTEX, Kanagawa, Japan) on days 7, 14, 21 and 31 after the surgery. X‐ray images were scored independently by two senior orthopaedic surgeons as previously described.^1^ In addition, the external callus width (CW) was assessed according to digitized lateral view radiographs. Briefly, the CW was measured by subtracting the maximum outer diameter of the external mineralized callus by the width of cortical bone.

Moreover, on post‐operative day 14, the fractured femurs were scanned using a Quantum FX micro–CT system (PerkinElmer, Waltham, MA, USA) at 1024 views, 16 frames per view, 90 kV and 88 mA. The callus volume of interest for the fractured bone was determined and the volume calculated with Analyze 12.0 software (PerkinElmer).

#### 2.3.3 Micro‐CT analysis of angiogenesis of the fracture sites

The vascularity of the fracture callus was assessed using a micro‐CT‐based method on post‐operative day 14.^1^ In brief, the whole vascular system was flushed through injecting heparinized (100 units/mL) normal saline, and a radiopaque contrast agent based on lead chromate (Flow Tech, Carver, MN, USA) was perfused by intracardiac injection. Next, animals were maintained at 4°C for 24 hours for compound polymerization. The femurs were preserved at 4°C for 2 days in 4% paraformaldehyde, soaked for 21 days in 10% ethylenediaminetetraacetic acid (EDTA) solution for decalcification, and finally placed in 4% paraformaldehyde after thorough washing with water. Specimens were scanned using the Quantum FX micro–CT system at 2400 views, 5 frames per view, 37 kV and 121 mA. Three‐dimensional images of the radiopaque contrast‐filled vascular network were assessed using Analyze 12.0 software.

#### 2.3.4 Histological analysis

Femurs were dissected on day 14 after the operation. Decalcified specimens with the entire callus were further processed to obtain paraffin‐embedded parts with a thickness of 6 μm. Immunohistochemical staining was performed as previously described.^34^ Specimens were incubated with rabbit anti‐CD31 antibodies (Abcam, Cambridge, UK, 1:50) at 4°C for 24 hours. The number of CD31‐positive vessels was counted in random areas around the fracture callus. The cross‐sectional vessel area was measured using ImageJ software (National Institutes of Health, Bethesda, MD, USA), as previously described.^35^

#### 2.3.5 Biomechanical testing

The animals were sacrificed after 14 days, and the osteotomized femora were explanted. Biomechanical testing was performed using a quasistatic 3‐point bending test. Briefly, after removing the intramedullary needle, the distal end of each femur was embedded in a cylinder using polymethyl methacrylate (Technovit 4000, Heraeus Kulzer GmbH, Wertheim, Germany), which was fixed in a hinge joint, and the proximal bone end was rested on the bending support. A bending load F on top of the callus tissue was applied at a crosshead speed of 1.5 mm/min until fracture. The stiffness was calculated from the slope of the force deflection curve. The bending stiffness and maximum load were measured and expressed as N/mm and N, respectively.

### 2.4 In vitro studies

#### 2.4.1 Primary osteoblast and endothelial cell culture

Primary osteoblasts were obtained from neonatal mice calvaria as previously described.^36^ Briefly, calvaria were harvested, and the periosteum, dura mater and all non‐osseous tissue were meticulously removed. Calvaria were washed with serial dilutions of Betadine in PBS, and osteoblasts were released by sequential digestion with 0.1% collagenase A and 0.2% dispase. Osteoblastic cells from 2‐5 fractions were pooled and seeded at a density of 2.5 × 10^4^ cells/cm^2^, and cells were maintained in DMEM plus 10% FBS at 37°C in an atmosphere containing 5% CO_2_. The media were replaced every other day, and osteoblasts in passage 2 were used for experiments. HUVECs were maintained in subconfluent cultures in Endothelial Cell Grow Medium‐2 (EGM‐2; Cambrex Bioscience, Walkersville, MD, USA). Cells from the fourth passage were used in the studies.

#### 2.4.2 Total RNA isolation and quantitative real‐time polymerase chain reaction (qRT‐PCR) of mRNA

Total RNA was obtained from cell samples using TRIzol reagent (Invitrogen, Carlsbad, CA, USA) according to the manufacturer's instructions. Detection and quality control of the RNAs were performed using a NanoDrop‐2000 spectrophotometer (Thermo Fisher Scientific, Waltham, MA, USA). cDNA was synthesized using a thermocycler (Applied Biosystems, Foster City, CA, USA) after mixing 20 μL of RNA and a master mix prepared with the High‐Capacity cDNA Synthesis Reverse Transcription kit (Applied Biosystems). RT‐PCR was conducted using a SYBR® Green PCR Master Mix (Applied Biosystems) according to the manufacturer's protocol. The PCR cycling conditions were 95°C for 10 minutes followed by 40 cycles of 95°C for 15 s and 60°C for 1 minute. All gene expression data were normalized against the values for glyceraldehyde 3‐phosphate dehydrogenase (GAPDH).

#### 2.4.3 Exosome uptake by HUVECs

To determine uMSC‐Exos uptake by HUVECs, exosomes were labelled with a green fluorescent dye (PKH67; Sigma‐Aldrich, St. Louis, MO, USA) as previously described^37^ and later incubated with HUVECs at 37°C for 3 hours. These cells were subsequently washed with PBS and fixed in 4% paraformaldehyde for 15 minutes. Fixed cells were washed with PBS, and nuclei were stained with DAPI (0.5 μg/mL; Invitrogen). Fluorescence microscopy was applied to detect the green signals in cells.

#### 2.4.4 Cell cycle analysis using flow cytometry

Target cells were harvested, centrifuged and washed 3 times with PBS. Next, the cells were suspended and fixed in 70% ethanol at 4°C for 24 hours. After washing twice with PBS, fixed cells were resuspended in 500 μL PBS containing 50 μg/mL PI, 100 μg/mL RNase A, 0.2% Triton X‐100 and incubated for 30 minutes.^31^ Next, the cell cycle was tested by flow cytometry (Beckman Coulter, Brea, CA, USA).

#### 2.4.5 EdU incorporation and immunofluorescence staining

To determine the proliferation ability of target cells, cells were labelled with EdU and stained as previously described.^38^ Briefly, 1 × 10^5^ cells were seeded in DMEM supplemented with 10% FBS, and a 1:1000 dilution of EdU‐labeling reagent (Invitrogen) was added after 24 hours. Forty‐eight hours later, the cells were fixed with methanol, incubated in 3% bovine serum albumin (BSA) in PBS, and subsequently incubated in 0.5% Triton® X‐100 in PBS at room temperature for 20 minutes. The cells were detected with a Click‐iT Edu Alexa Fluor 555 Imaging Kit (Invitrogen) according to the manufacturer's protocol. The cells were counterstained with 4′,6‐diamidino‐2‐phenylindole (DAPI, Sigma‐Aldrich, St. Louis, MO, USA) and later mounted in standard mounting media. Images were analysed using MacBiophotonics ImageJ software (National Institutes of Health).

#### 2.4.6 Scratch wound assay

Migration was evaluated by scratching a confluent layer of HUVECs in a 24‐well plate using a P200 pipette tip. After a PBS wash to remove loose cells, 200 µL of test medium was added, and the plate was incubated at 37°C. Images were obtained at 0, 12 and 24 hours, after which the reduction in the wound area was determined using Image‐Pro Plus software (Media Cybernetics, Rockville, MD, USA). Cells from three random fields were counted.

#### 2.4.7 Tube formation assay

Basement membrane matrix (Matrigel, BD Biosciences, Bedford, MA, USA) was dissolved overnight at 4°C, and 48‐well plates were prepared with 100 μL of Matrigel in each well. After coating and incubating the loaded wells at 37°C overnight, 3 × 10^4 ^HUVECs were cultured in 200 μL of medium comprising 50% EGM‐2 medium and 50% conditioned medium.^39^ Tube branches and total tube length were calculated using MacBiophotonics ImageJ software (National Institutes of Health).

#### 2.4.8 Western blot analysis

Fifty‐one proteins were resolved by SDS‐polyacrylamide gel electrophoresis and moved to Immobilon Polyvinylidene difluoride membranes (Millipore, Billerica, MA, USA). At room temperature, blots were blocked with 4% BSA for 1 hour and probed with rabbit anti‐human antibody against HIF‐1α (1:1000) for 1 hour. After three washes, the blots were incubated for 1 hour at room temperature with donkey anti‐rabbit peroxidase‐conjugated secondary antibody (1:3000) and visualized by enhanced chemiluminescence using Kodak X‐OMAT LS film (Eastman Kodak, Rochester, NY, USA).^40^

### 2.5 Statistical analysis

All the experiments were performed in triplicate, and the average data of three independent experiments were presented. The data are presented as the mean ± standard deviation (*SD*). The radiographic scores were analysed using the Mann‐Whitney *U* test. Comparisons between samples were conducted using Student's *t* test; for more than two groups, we used one‐way analysis of variance with the Bonferroni method. *P* < 0.05 was considered to be statistically significant.

## 3 RESULTS

### 3.1 Characterization of exosomes

**Figure 1:**
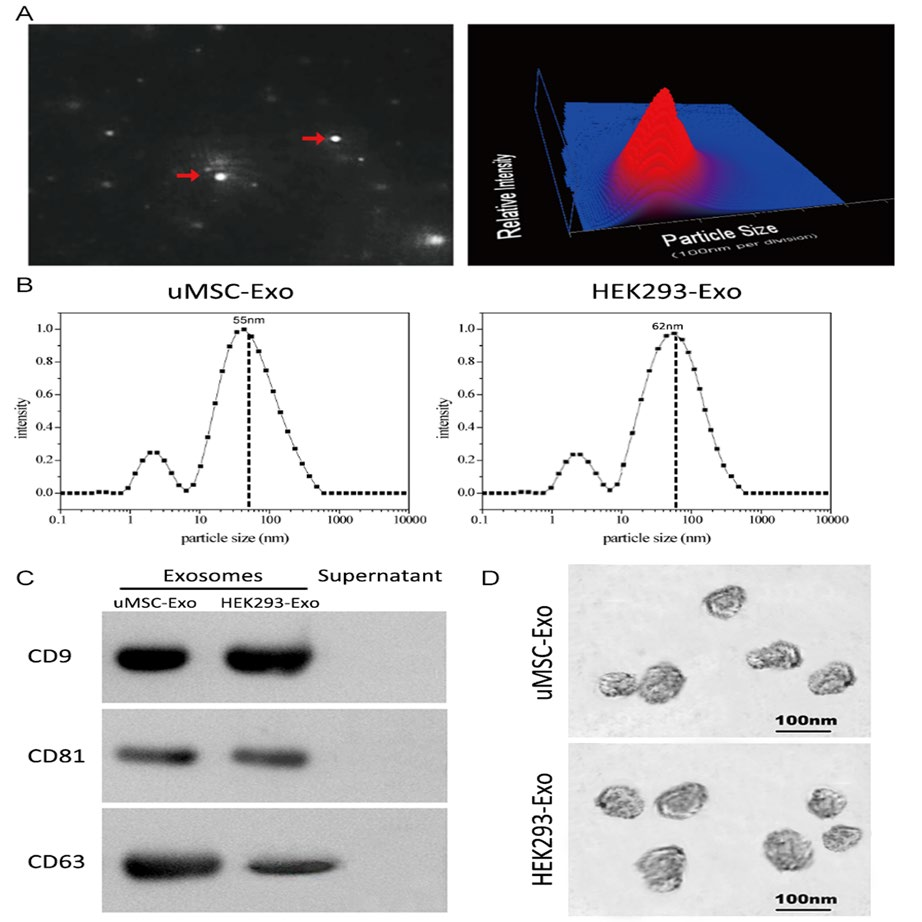
Characterization of exosomes derived from human umbilical cord‐derived mesenchymal stem cells (uMSCs) and HEK293 cells. A, Representative image of purified exosome particles (left panel) and the particle size distribution in purified uMSC‐Exo (right panel) as determined by NanoSight. The red arrow indicates exosomes. B, Precise particle size distribution of purified uMSC‐Exo and HEK293‐Exo measured by laser light scattering spectrometer. The dashed dot line indicates the peak particle size of purified exosomes. C, Western blot analysis of the exosomes surface markers. D, Morphology of the exosomes observed by TEM

NanoSight, TEM, a laser light scattering spectrometer, and Western blotting were used to characterize the purified nanocarriers. The results showed that the vast majority of these particles were approximately 100 nm and spherical, and expressing the characteristic surface markers, including CD9, CD63 and CD81, all of which validated successful exosomes collection and purification (Figure 1A‐D).

### 3.2 uMSC‐Exos enhanced bone healing and angiogenesis at the site of femur fracture in vivo

#### 3.2.1 X‐ray analysis of fracture callus formation

**Figure 2:**
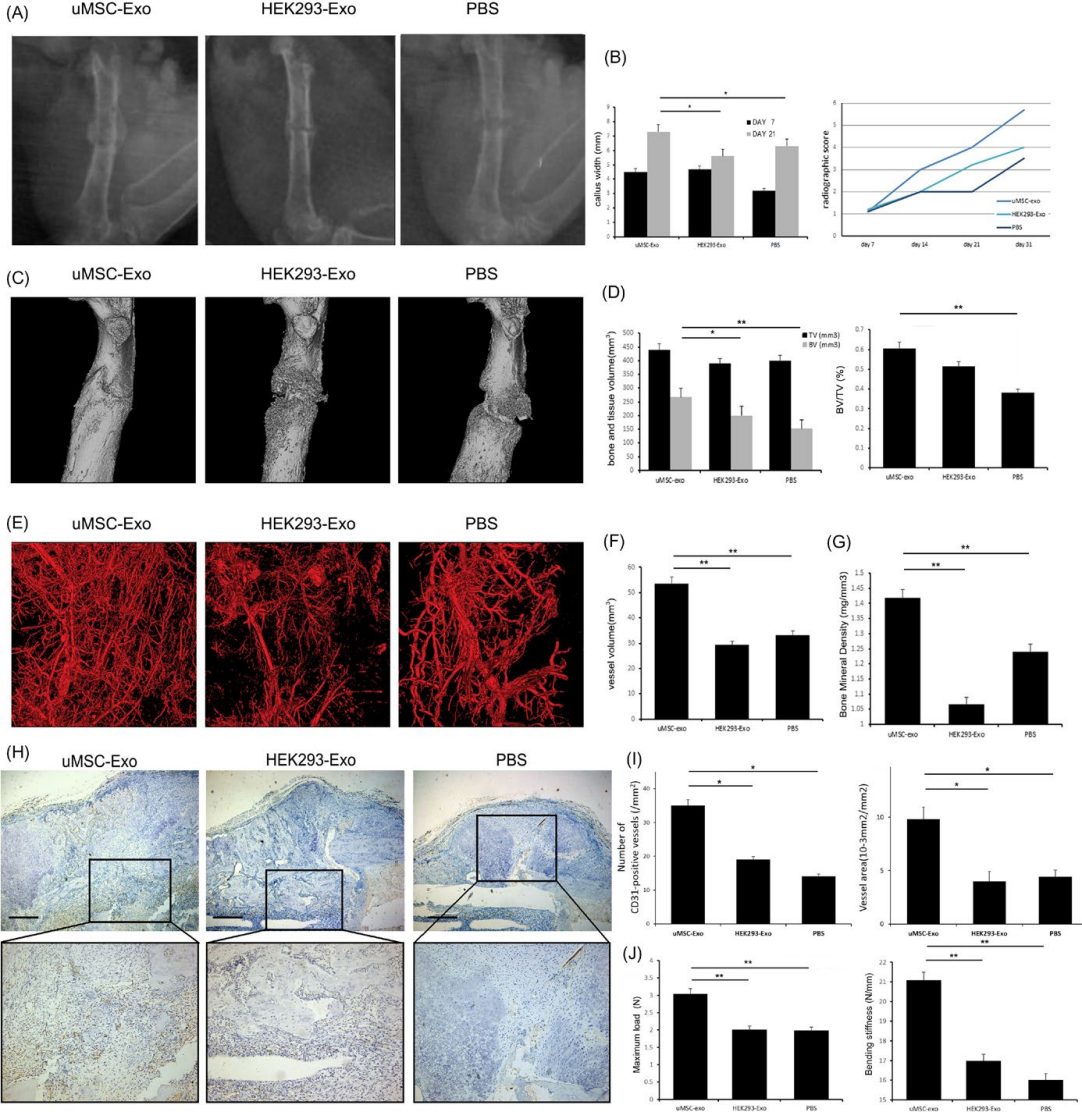
Radiographic and histological analysis of the fracture healing. A, Representative X‐ray images of the fractures on post‐operative day 14. B, Quantitative analysis of the CW on post‐operative days 7 and 21 (left panel). Bone formation in X‐ray images was assessed on post‐operative days 7, 14, 21 and 31 (right panel), using a radiographic score as described in Section 2. n = 6. C, Representative Micro‐CT images of the fractured femur on post‐operative day 14. D, TV and BV of the callus, and BV/TV on post‐operative day 14 was quantified. n = 6. E, BMD on post‐operative day 14 was quantified using Micro‐CT. n = 6. F, Representative Micro‐CT images of the vascular system on post‐operative day 14. G, On day 14 after surgery, vessel volume was quantified on Micro‐CT images. n = 6. H, The fractured callus on post‐operative day 14 stained with anti‐CD31. Representatives were shown, and boxed areas were enlarged on the bottom. Scale bar for original images = 200 mm. I, The number of CD31‐positive vessels (left panel) was counted, and the ratio of vessel area (right panel) was measured n = 6. (*P < 0.05, **P < 0.01, CT, computed tomography; BMD, bone mineral density; BV, bone volume; TV, total volume

The bone formation in femoral fracture healing was radiographically evaluated. A hard callus with bridging of the fracture gap was observed in X‐ray images, and the fracture gap was obvious on post‐operative day 14 in all three groups. The border between the newly formed hard callus and the existing cortical bone had disappeared with the observed remodelling processes. However, the uMSC‐Exo group had larger callus volumes than the other two groups (Figure 2A). Quantitative analysis of the CW showed significantly higher values in the uMSC‐Exo group than those of the HEK293‐Exo and PBS groups on post‐operative day 21 (Figure 2B). Bone formation based on X‐ray images was evaluated using a radiographic score as described previously,^1^ and a significant difference between the uMSC‐Exo group and the other groups was observed on post‐operative days 14, 21 and 31 (Figure 2B).

#### 3.2.2 Micro‐CT analysis of bone regeneration and angiogenesis

High‐resolution micro‐CT scanning was performed and reconstructed to qualitatively evaluate callus and vessel volumes on post‐operative day 14 (Figure 2C). Similar to the earlier results, the administration of uMSC‐Exos led to a statistically significant increase in the bone mineral density (BMD), bone volume (BV) and BV/TV (Figure 2D,E).

Vascular growth within the fracture callus was evaluated by imaging of contrast‐perfused, decalcified specimens on post‐operative day 14 (Figure 2F). The vessel volume was remarkably increased in experimental group (Figure 2G).

#### 3.2.3 Histological analysis

Immunohistochemistry was used to observe the tissues surrounding the femoral fracture callus on post‐operative day 14 (Figure 2H). Results showed that there were significantly more CD31‐positive blood vessels after surgery by the administration of uMSC‐Exos (Figure 2I). Compared with control groups, the ratio of the vessel areas was also higher in the uMSC‐Exo group (Figure 2I).

#### 3.2.4 Biomechanical analysis

On post‐operative day 14, biomechanical analysis showed an enhanced maximum load at failure and bending stiffness in the uMSC‐Exo‐treated group compared with both control groups (Figure 2J).

### 3.3 Expression levels of osteogenesis‐ or angiogenesis‐related genes in target cells stimulated by uMSC‐Exos

**Figure 3:**
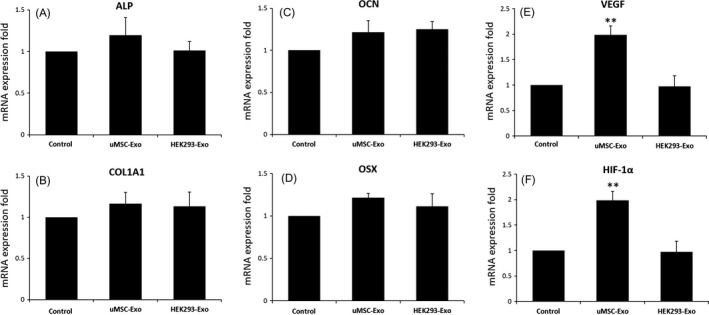
The expression levels of selected differentially expressed genes in target cells on 14 day after uMSC‐Exo treatment were quantified by qRT‐PCR. A‐D, Total RNA was extracted, and the expression levels of osteogenesis‐related genes in primary osteoblasts were analysed by qRT‐PCR. n = 3, **P < 0.01. E, F, Total RNA was extracted, and the expression levels of angiogenesis‐related genes in HUVECs were analysed by qRT‐PCR. n = 3, **P < 0.01

We added equal quantities of uMSC‐Exos, HEK293‐Exos or PBS to the primary osteoblasts, and incubated the cells in differentiation medium for 14 days. The final mRNA expression levels of OSX, OCN, COL1A1 and ALP in osteoblasts were obtained and did not show significant differences among 3 groups (Figure 3A), indicating that uMSC‐Exos did not regulate the expression of osteogenesis‐related genes in osteogenic induction medium. HUVECs were also similarly processed. The mRNA expression levels of both VEGF and HIF‐1α in HUVECs were detected, and both exhibited remarkable increases after uMSC‐Exos treatment (Figure 3B).

### 3.4 Pro‐angiogenesis effects of uMSC‐Exos on HUVECs

**Figure 4:**
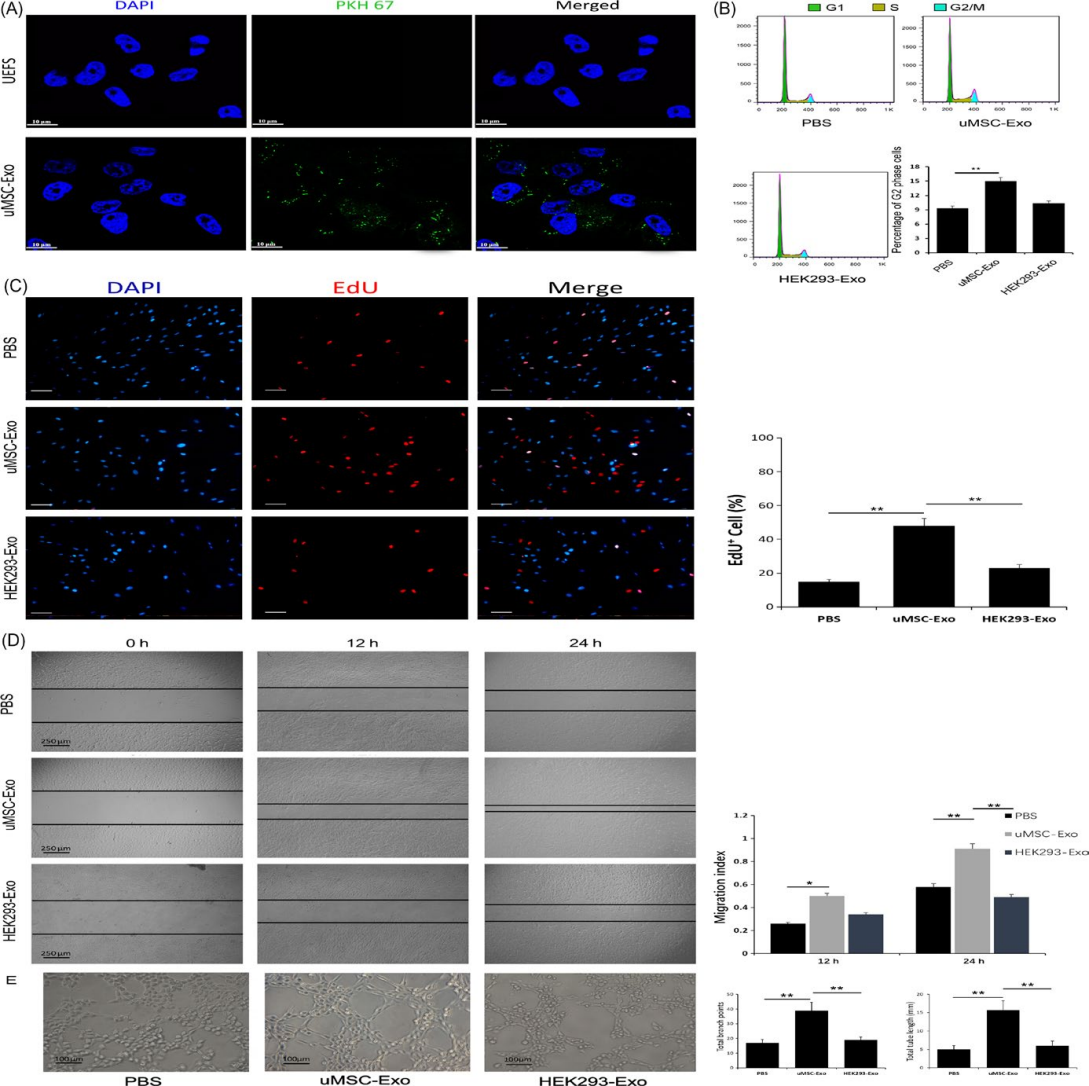
Internalization of uMSC‐Exos in HUVECs, and their pro‐angiogenesis effects on the recipient cells. A, Fluorescence microscopy analysis of PKH67‐labelled uMSC‐Exos internalization by HUVECs. The green‐labelled exosomes were visible in the perinuclear region of recipient cells. Scale bar: 50 μm. B, Cell cycle assay of differently treated HUVECs. Representative images were shown in the left. The percentage of G2 population was shown in the right panels. n = 3, **P < 0.01. C, Cells were dispersed, fixed and stained for DAPI and EdU (DAPI and EdU are indicated by blue and red staining, respectively). EdU incorporation for different treatments was visualized using a fluorescence microscope (left panel). Scale bar: 50 μm. The percentage of EdU‐positive (proliferating) cells for each treatment was quantitated using ImageJ software (right panel). n = 3, **P < 0.01. D, The migration ability of HUVECs in different treatment groups was tested by the scratch wound assay. Representative images were shown in the left panels. Scale bars = 250 μm. Quantitative analysis of the migration rates was shown in the right panels. n = 3, *P < 0.05, **P < 0.01. E, uMSC‐Exos stimulated the tube formation ability of HUVECs. Representative images were shown in the left panels. Scale bar: 100 μm. Quantitative analysis was shown in the right panels. n = 3, **P < 0.01

To further determine the roles of uMSC‐Exos in angiogenesis, at first, we verified the internalization ability of our purified exosomes using the PKH67 assay. After staining, washed and ultracentrifuged uMSC‐Exos were added to HUVECs. Fluorescence microscopy analysis indicated that cells administrated with stained uMSC‐Exos presented prominent PKH67 fluorescence located in the cytoplasm (Figure 4A), while the uMSC‐Exos‐free supernatant (UEFS, the concentrated medium left after exosome removal) group showed no obvious fluorescence. This result revealed that our purified exosomes did have cellular transmission activity.

Next, endothelial cell proliferation and migration capabilities, which are crucial in the process of angiogenesis, were also assessed by cell cycle analysis, EdU test and scratch wound assay. As shown in Figure 4B, the percentage of cells in the G2 phase was higher in the uMSC‐Exo group than that in the control groups. Consistent with the results of cell cycle analysis, the EdU test demonstrated that, compared with control groups, there was a remarkably higher percentage of EdU‐positive (proliferating) cells in the uMSC‐Exo group (Figure 4C). The migration ability of HUVECs stimulated by uMSC‐Exos was also significantly enhanced, while no obvious promotion was observed in the control groups (Figure 4D).

Tube formation reflects terminal aspects of blood vessel formation and is a critical step in angiogenesis.^41^ Finally, the tube formation assay in HUVECs indicated that, in comparison with HEK293‐Exos or PBS, uMSC‐Exos significantly enhanced the angiogenic tube formation ability of endothelial cells (Figure 4E).

Therefore, we believe that the positive effects of uMSC‐Exos in promoting fracture healing may not rely on accelerating osteogenetic differentiation but depend on certain molecular or cellular regulations during angiogenesis.

### 3.5 HIF‐1α is required in uMSC‐Exo‐enhanced VEGF expression and angiogenesis

**Figure 5:**
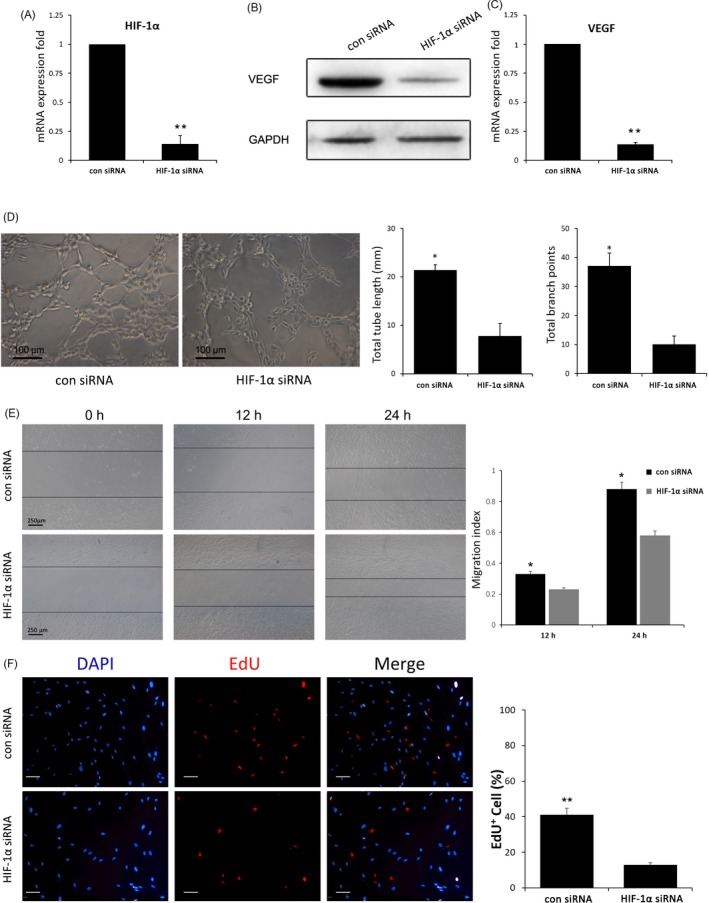
Regulating the expression of HIF‐1α in HUVECs stimulated with uMSC‐Exos‐mediated VEGF expression and angiogenesis ability. A, The inhibitory efficiency of the siRNA targeting HIF‐1α was verified by qRT‐PCR. n = 3, **P < 0.01. B, Western blot analysis showed the inhibition of HIF‐1α decreased the VEGF protein in HUVECs stimulated with uMSC‐Exos. C, qRT‐PCR analysis indicated that inhibition of HIF‐1α decreased the mRNA expression level of VEGF in HUVECs stimulated with uMSC‐Exos. n = 3, **P < 0.01. D, Optical micrographs of tube formation assay of HIF‐1α‐regulated HUVECs stimulated with uMSC‐Exos were shown in the left panels. Scale bar: 100 μm. Quantitative analysis was shown in the right panels. *P < 0.05. E, The migration ability of HIF‐1α‐regulated HUVECs stimulated with uMSC‐Exos was tested by the scratch wound assay. Representative images were shown in the left panels. Scale bars = 250 μm. Quantitative analysis of the migration rates was shown in the right panels. n = 3, *P < 0.05. F, The proliferation ability of HIF‐1α‐regulated HUVECs stimulated with uMSC‐Exos was tested by the EdU test (left panel). Scale bar: 50 μm. The percentage of EdU‐positive (proliferating) cells for each treatment was quantitated using ImageJ software (right panel). n = 3, **P < 0.01

It has been reported that HIF‐1α can regulate the expression of VEGF.^42^ To determine whether HIF‐1α is an essential molecule in mediating uMSC‐Exo‐induced VEGF mRNA expression and angiogenesis, lentivirus vectors carrying either HIF‐1α siRNA or scrambled siRNA (con siRNA) were constructed and used to transfect HUVECs. qRT‐PCR analysis revealed that HIF‐1α siRNA led to remarkable suppression of HIF‐1α expression compared to that in con‐siRNA group, which verified the inhibitory efficiency (Figure 5A). Then, HUVECs transfected with HIF‐1α siRNA or scrambled siRNA were administered with uMSC‐Exos, after which the angiogenic capabilities of HUVECs were assessed. The results suggested that inhibition of HIF‐1α in HUVECs significantly decreases VEGF expression, while promoted VEGF expression was observed in HUVECs transfected with scrambled siRNA (Figure 5B‐C), demonstrating that HIF‐1α was an important regulator for uMSC‐Exo‐induced VEGF expression in the cells.

The angiogenic capabilities were evaluated with tube formation, EdU and scratch wound assays. Representative images of tube formation assay and quantitative analysis indicated that HUVECs with scrambled siRNA formed significantly more capillary‐like branches, and the total tube length of the capillary‐like structures was considerably longer than that of HIF‐1α‐silenced HUVECs (Figure 5D). Scratch wound assay showed that HUVECs infected with scrambled siRNA exhibited markedly enhanced motility, as determined by the migration index (Figure 5E). In terms of the EdU test, there was a remarkably higher percentage of EdU‐positive (proliferating) cells in con‐siRNA group (Figure 5F). These results suggested that regulating HIF‐1α expression is one of the potential mechanisms in bone repair with uMSC‐Exos.

## 4 DISCUSSION

In the past several decades, MSC‐based therapies have been demonstrated to trigger a complicated process of interactions among numerous types of cells, components of the extracellular matrix, and signalling molecules following injury. Such therapies have been reported to promote bone regeneration and fracture healing.^4^, ^5^ However, the mechanism by which this effect occurs has not been thoroughly elucidated to date. Among numerous potential molecular mechanisms, exosomes released from MSCs have been regarded as critical and have attracted increasing attention.^9^, ^10^

Exosomes, one type of extracellular vesicles, are membrane vesicles measuring approximately 40‐100 nm in diameter that contain functional proteins, mRNAs and miRNAs, which can be transferred between cells and thus regulate cellular activities of recipient cells.^43^ As important mediators of intercellular communication with a cell‐specific ability to dock and unload their cargo, they represent a novel method for cell‐free regenerative medicine.^19^, ^20^ Positive effects of various‐sources exosomes in bone regeneration have been demonstrated in animal or in vitro experiments.^31^, ^44^, ^45^ Raghuvaran Narayanan et al^46^ discovered that exosomes from human marrow stromal cells (HMSCs) could trigger osteogenic differentiation of undifferentiated HMSCs both in vitro and in vivo. In addition, they observed an increase in the expression levels of VEGF after treatment with exosomes, indicating the potential of the exosome to promote vascularization in the fracture healing process. Yunhao Qin et al^31^ proved that BMSC‐derived exosomes could also stimulate osteoblastic differentiation via the increased expression of osteogenic genes in vitro. Moreover, an in vivo investigation demonstrated that BMSC‐derived exosomes took effects in bone regeneration with rats of critical‐sized calvarial bone defects. Researchers further explored the potential molecular mechanism and implied that microRNAs contained in exosomes played a critical role in the enhanced healing process.

Among the available sources of MSCs, the umbilical cord represents a cost‐effective, productive, feasible, acceptable and universal source from which to isolate MSCs.^25^ Studies have indicated that uMSCs could improve recovery in diverse animal models of limb ischaemia,^47^ endometrial injury,^48^ diabetic wound^49^ and ischaemic/reperfusion injury^50^ through promoting angiogenesis. Additionally, Todeschi MR et al^28^ discovered that uMSCs were also capable of promoting bone formation via an indirect pro‐angiogenic effect rather than a direct differentiation of the implanted cells. As for the underlying mechanism for pro‐angiogenesis, they demonstrated the role of the uMSC secretome as a result of paracrine functions, but they could not confirm that an intercellular cross talk between the exogenous stem cells and the host endogenous progenitors also occurred.

Our current study provides the first strong evidence that an intercellular cross talk between the exogenous stem cells and the host endogenous progenitors via exosomes occurs in fracture healing. The in vivo results suggested that uMSC‐Exos could strongly enhance angiogenesis and bone regeneration. In vitro, the expression levels of osteogenesis‐ or angiogenesis‐related genes in target cells stimulated by uMSC‐Exos were analysed by RT‐PCR. ALP, OCN, COL1A1 and OSX mRNA expression levels were not significantly different while VEGF and HIF‐1α were remarkedly increased. Combining the subsequent findings that the abilities of HUVECs stimulated by uMSC‐Exos in proliferation, migration and tube formation were significantly enhanced, it could be inferred that the uMSC‐Exo‐enhanced fracture healing may not rely on accelerating osteogenetic differentiation but depend on pro‐angiogenesis.

Angiogenesis is an essential component of fracture healing, and defective angiogenesis at the fracture site commonly leads to poor outcomes.^51^, ^52^ One of the main players in angiogenesis in fracture healing is the endothelial cells,^53^, ^54^ which proliferate, migrate, form tubes and finally produce a non‐leaky conduit where blood flows. This process must be properly coordinated in time and space, regulated by sophisticated mechanisms with diverse molecules. Among these, HIF‐1α driving the expression of VEGF, which is a well characterized key factor in angiogenesis, has been reported important.^23^, ^56^ To identify if this mechanism also plays a major role in the pro‐angiogenesis process with uMSC‐Exos, we further used specific RNA inhibitors or siRNAs, and discovered it did regulate the expression of VEGF and the angiogenic ability of endothelial cells, which may be one of the underlying mechanisms of promoted bone regeneration with exosomes.

In summary, our current study showed that uMSC‐derived exosomes could accelerate the proliferation, migration and tube formation of endothelial cells, further promoting angiogenesis and ultimately enhancing fracture healing. Exosomes, as an intercellular communicator upregulating HIF‐1α and controlling VEGF gene expression of target cells, may be one of the underlying mechanisms in the promoted process. uMSC‐Exos play a direct role and participate in a novel mechanism in uMSC‐based bone fracture therapy. This information may be useful to develop a new cell‐free therapy for bone unions in the future.

## CONFLICT OF INTEREST

All authors state that they have no conflicts of interest.
